# Clinical Response to Venetoclax and Decitabine in Acute Promyelocytic Leukemia With a Novel RARA-THRAP3 Fusion: A Case Report

**DOI:** 10.3389/fonc.2022.828852

**Published:** 2022-02-07

**Authors:** Baoquan Song, Xin Wang, Xin Kong, Man Wang, Li Yao, Hongjie Shen, Jian Zhang, Huiying Qiu

**Affiliations:** ^1^ National Clinical Research Center for Hematologic Diseases, Jiangsu Institute of Hematology, The First Affiliated Hospital of Soochow University, Suzhou, China; ^2^ Institute of Blood and Marrow Transplantation, Collaborative Innovation Center of Hematology, Soochow University, Suzhou, China

**Keywords:** RARA-THRAP3, acute promyelocytic leukemia, venetoclax, decitabine, case report

## Abstract

Variant acute promyelocytic leukemia (APL) showed quite different aspects, and the current treatments remained challenged at present. Venetoclax, a selective inhibitor of B-cell lymphoma 2 (BCL-2), is a small molecule that has been studied in several hematologic malignancies as both monotherapy and in combination with other agents. However, there is little of its use in the treatment of APL or variant APL. In this report, we identified THRAP3 as novel RARA fusion in resembling APL, which was resistant to all-trans retinoic acid (ATRA) combined arsenic trioxide (ATO) chemotherapy. Then, the patient was salvaged by low-dose venetoclax and decitabine. The treatment in this case demonstrates the potential ability of venetoclax in variant APL, thus providing a new treatment option for all kinds of APL.

## Introduction

Acute promyelocytic leukemia (APL) is commonly associated with t(15;17) (q24.1;q21.2), which results in the fusion of PML-RARA, a translocation-driven fusion oncoprotein, which accounts for the disease response to all-trans retinoic acid (ATRA) and arsenic trioxide (ATO) (1, 2). To date, several other types of rare X-RARA fusions have been described. Unfortunately, most of the novel retinoic acid receptor fusions associated with APL are clinically resistant to ATRA or ATO (3, 4).

Venetoclax, an oral small-molecule B-cell lymphoma 2 (BCL-2) inhibitor, was approved by the FDA for front-line treatment of newly diagnosed AML in older patients or those unfit for induction chemotherapy (5). Although venetoclax has been used in the treatment of acute myeloid leukemia (AML) in recent years, there are only few reports of its use in the treatment of APL (6, 7).

Here, we report a novel RARA-THRAP3 fusion in an APL patient with insensitivity to ATRA and ATO therapy, who achieved complete remission following salvage therapy with combined low-dose venetoclax and decitabine.

## Case Presentation

**Figure 1:**
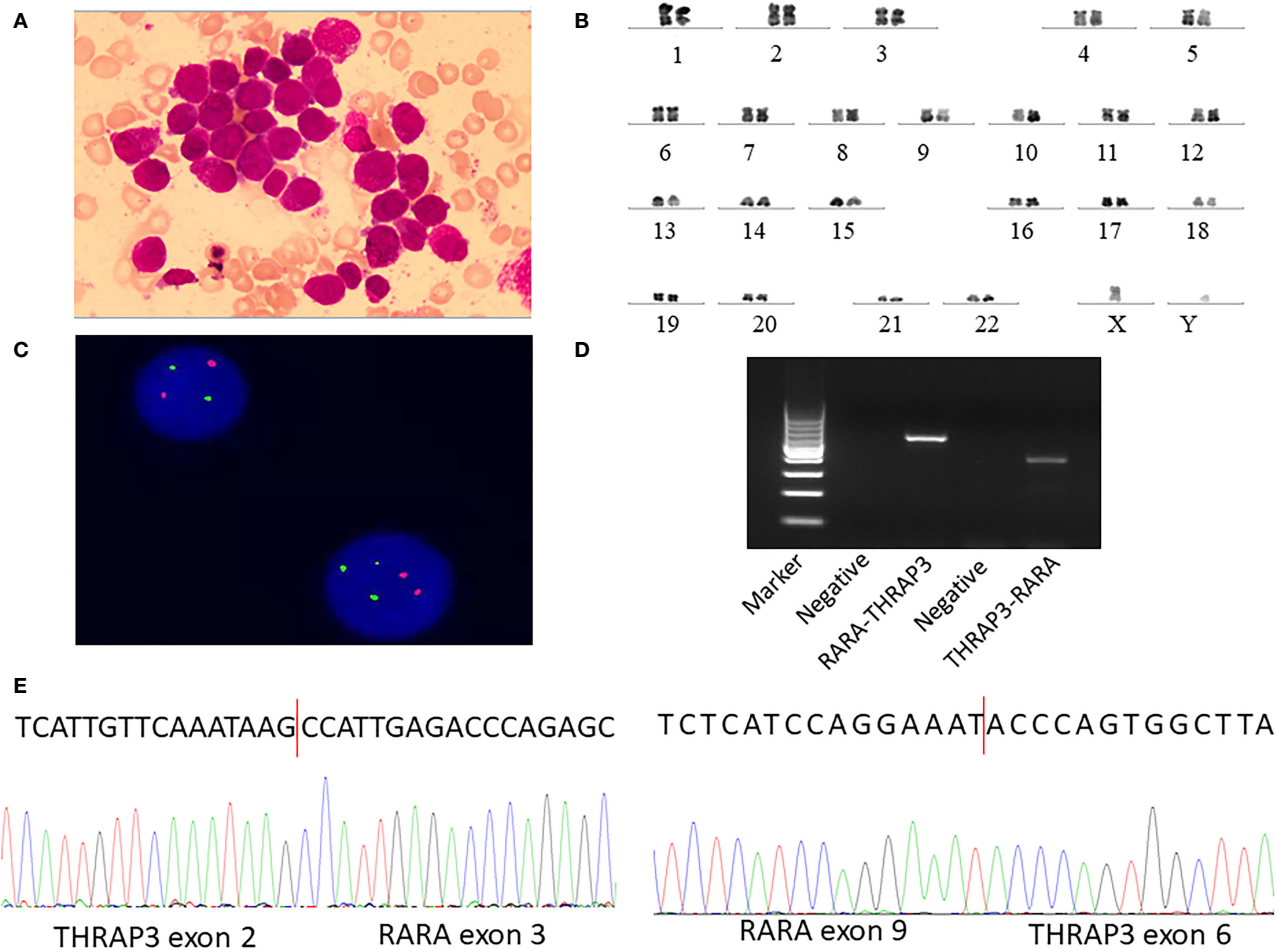
Identification of novel recurrent fusions RARA-THRAP3 in APL lacking t(15;17) (q22;q12)/PML-RARA. **(A)** Several promyelocytes, with hypergranulated cytoplasm and invaginated nuclei, are shown in the diagnostic bone marrow (BM) aspirate (Wright–Giemsa stain, ×1,000). **(B)** A karyotype performed on the diagnostic BM revealed 46, XY [20]. **(C)** Fluorescence *in situ* hybridization using RARA dual color probes found RARA rearrangement or the split RARA signals. Intact RARA are shown as (red) and (green), while the abnormal RARA signals are indicated as (yellow). **(D)** Electrophoresis of RT-PCR products from the patient showed the RARA-THRAP3 fusion transcript, whereas the reciprocal THRAP3-RARA transcript were also detected. **(E)** Partial nucleotide sequences surrounding the junctions of the RARA-THRAP3 fusion transcript. The RARA-THRAP3 fusion transcript was a fusion of THRAP3 exon 9 and RARA exon 6 genes. The THRAP3-RARA fusion transcript was a fusion of THRAP3 exon 2 and RARA exon 3 genes.

**Figure 2:**
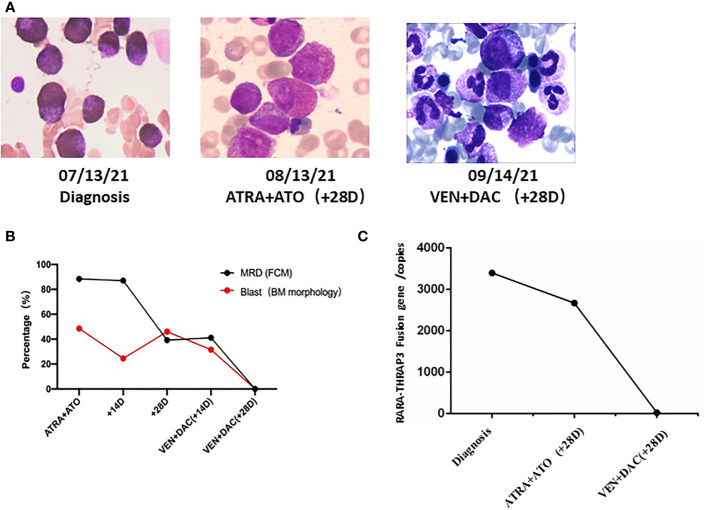
The novel recurrent RARA-THRAP3 fusion APL patient was salvaged by low-dose venetoclax combined decitabine. **(A)** Timeline of treatment and bone marrow blast percentages for patient (×1,000). **(B)** Flow cytometric immunophenotyping of primary diagnosis. **(C)** RARA-THRAP3 fusion gene was detected by RT-PCR. BM, bone marrow; FCM, flow cytometry; MRD, minimal residual disease; DAC, decitabine; VEN, venetoclax.

A 34-year-old man was admitted to our hospital on July 13, 2021, because of abdominal pain and fever for 1 week. Peripheral blood examination results revealed a white blood cell count of 1.53 × 10^9^/l, a hemoglobin level of 116 g/l, and a platelet count of 137 × 10^9^/l. The bone marrow was hypercellular with 49.5% promyelocytes, frequent Auer rods, and a positive result for peroxidase stain (**Figure 1A**). The blasts were positive for CD13 and CD33 but negative for CD34, CD117, and HLA-DR by flow cytometry (**Table 1** and **Supplementary Figure 1A**). The patient was diagnosed with suspicion of APL. However, a normal activated partial prothrombin time (aPTT), prothrombin time (PT), and thrombin time were observed in addition to the decreased fibrinogen level (1.0 g/l, reference, 2.00–4.00 g/l). Due to clinical suspicion of APL, the combination of ATRA (20 mg/m^2^, BID) and arsenic trioxide (ATO) (10 mg, QD) immediately initiated. Subsequently, multiplex quantitative RT-PCR failed to detect PML-RARA fusion and chromosomal examination revealed a normal karyotype of 46, XY (20) (**Figure 1B**). No mutations were detected by next-generation DNA sequencing. Fluorescence *in situ* hybridization (FISH) detects the rearrangement or amplification involving the RARA gene; however, it did not show its gene partners (**Figure 1C**). After a 2-week combination treatment of ATRA and ATO, bone marrow (BM) smear showed hypercellularity with 24.5% hypergranular promyelocytes (**Supplementary Figure 1B**), considering that the treatment is partially effective. Frustratingly, follow-up BM 28 days after ATRA and ATO therapy showed a persistently hypercellular marrow with 46% hypergranular promyelocytes (**Figures 2A, B** **)**.

**Table 1:** Clinical characteristics of the patient at baseline.

Patient	Sex/age (years)	Blast (%)	Immunophenotype	Cytogenetic	Molecular	FISH
WGL	Male/35	49.5%	CD13^+^, CD33^+^, CD45^dim^, CD34^-^, CD117^-^, HLA-DR^-^CD56^-^	46, XY [20]	Negative	RARA (rearrangement or amplification)

In order to characterize the abnormality involving the RARA gene, we performed next-generation RNA-sequencing (Illumina, Inc.) analysis of the BM samples and revealed a novel gene fusion event between THRAP3 and RARA, leading to the formation of a couple of reciprocal transcripts, THRAP3-RARA and RARA-THRAP3. In THRAP3-RARA, THRAP3 exon 2 was fused in-frame to RARA exon 3, whereas in RARA-THRAP3, RARA exon 9 was fused in-frame to THRAP3 exon 6 (**Supplementary Figures 2A, B** **)**. For validation of this novel fusion, we performed RT-PCR and Sanger sequencing and confirmed the THRAP3-RARA and RARA-THRAP3 fusion transcript. The following primers were designed to amplify THRAP3-RARA mRNA: forward (at THRAP3 exon 3), 5′-TTGTAAAGTGAAGAAGCCAGTG-3′, and reverse (at RARA exon 4), 5′-TCCTTGGACATGCCCACT-3′. The following primers were used to amplify RARA-THRAP3 mRNA: forward (at RARA exon 7), 5′-ACACCATGACCTTCTCGGAC-3′, and reverse (at THRAP3 exon 6), 5′-GAGGGGCTACGCTGAGACTG-3′. The expected bands of approximately 339 bp (THRAP3-RARA) and 520 bp (RARA-THRAP3) were visualized by electrophoresis (**Figure 1D**), and the PCR product was analyzed by Sanger sequencing and confirmed THRAP3-RARA and RARA-THRAP3 fusion transcripts (**Figure 1E**) in the patient’s cells, which can be completely aligned to that of RNA-seq. This confirmed the variant case of APL. For THRAP3-RARA fusion, the break point of THRAP3 occurred on the 5′ untranslated region (5′UTR), which was an out-of-frame transcript that could not express the fusion protein. The following analysis of the fusion gene focuses on RARA-THRAP3.

However, additional intense chemotherapy could not be given due to persistent cytopenias, unexplained fever, and a poor physical status (ECOG performance status greater than 2). The patient received re-induction therapy with a cycle venetoclax (venetoclax 50 mg Days 1–14, 100 mg Days 14–28) combined low-dose decitabine (10 mg Days 1–10) regimen. There were no clinically significant tumor lysis syndrome and adverse events during treatment. BM aspirate at day 28 demonstrated morphologic CR, and a negative minimal residual disease (MRD 2.3 × 10^-4^) (**Figures 2A, B** **)**. The patient also achieved molecular remission by detection of RARA-THRAP3 fusion transcripts by RT-qPCR (**Figure 2C**). Subsequently, the patient underwent consolidation with 2 courses of intermediate-dose cytarabine (1.0 g/m^2^ 12-h iv infusion for 3 h on Days 1–3) and idarubicin (10 mg/m^2^ day iv infusion on days 3–5) successfully, and CR was still maintained at now 5 months. The patient is currently undergoing HLA-matched unrelated donor hematopoietic stem cell transplantation (URD-HSCT) with a myeloablative conditioning regimen.

## Discussion

Variant APLs often showed similar typical morphological; however, the genetic landscape, their pathogenesis, and treatment were quite different. To date, more than 17 diverse translocations involving RARA (X-RARA) have been found in rare leukemia patients (3, 8). Meanwhile, RARB rearrangements, RARG rearrangements, and other genetic events have been associated with very rare cases of APL (9, 10). Due to the abnormal molecular characteristics of partners, most of the variant APLs are clinically resistant to ATRA or ATO. In clinical work, how to recognize, diagnose, and treat variant APL remained still challenged at present.

Venetoclax, the Bcl-2 inhibitor, is a small molecule that has shown promising therapeutic effects in AML and lymphatic malignancies. Due to its effect on the restoration of the apoptotic pathway, venetoclax as a targeted drug with a novel mechanism of action that has already demonstrated highly promising activity in others hematological malignancies such as multiple myeloma, non-Hodgkin lymphoma, myelodysplastic syndrome, and early T-cell lymphoblastic leukemia (11, 12). Interestingly, Liu et al. reported a novel HNRNPC-RARA fusion in APL lacking PML-RARA rearrangement, sensitive to venetoclax-based therapy. Moreover, Abboud et al. demonstrated that decitabine may be an efficacious and more tolerable alternative to elderly patient with low-risk AP therapy (13). The results give us some hints; combination therapy with venetoclax and decitabine may show a promising clinical efficacy in variant APLs.

Thyroid hormone receptor-associated protein 3 (THRAP3), a gene located at chromosome 1q3.4, showed a ubiquitous expression in lymph node (RPKM 27.3), thyroid (RPKM 26.7), and other tissues. THRAP3 as a subunit of the transcription regulatory complex TRAP/mediator is involved in precursor-mRNA alternative splicing and DNA damage response and is a transcriptional coregulator of PPARγ in adipocytes. In this case, we revealed the presence of the RARA-THRAP3 fusion through next-generation RNA sequencing analysis and confirmed it by RT-PCR. In addition, our case exhibited resistance to typical APL therapy, including ATRA and ATO. Although there are very few studies to try new treatment strategies for variant APL, current experience in the treatment of this situation is overwhelmingly based on case reports and based on our previous treatment experience and a case report from Liu et al. The patient was treated with the combination of low-dose venetoclax and decitabine. Surprisingly, the patient achieved a complete remission and a stable cytogenetic remission after one cycle therapy.

In summary, we identified RARA-THRAP3 as another recurrent novel RARA fusion in APL, which exhibited resistance to ATRA and ATO treatment. Venetoclax plus decitabine regimen could be an effective and tolerable treatment for variant APL especially when initial induction therapy fails. Further studies are needed to better understand the biological properties, especially its sensitivity to venetoclax or decitabine, of this new RARA-THRAP3 fusion protein.

## Data Availability Statement

The original contributions presented in the study are included in the article/**Supplementary Material**. Further inquiries can be directed to the corresponding authors.

## Ethics Statement

Written informed consent was obtained from the relevant individual(s), and/or minor(s)’ legal guardian/next of kin, for the publication of any potentially identifiable images or data included in this article.

## Author Contributions

HQ and BS contributed equally to this study and performed most of the experiments. JZ and Mengyun Li were the principal investigators. XK, Xiaoxia Wu, MW, and LY analyzed and discussed the data. All authors contributed to the article and approved the submitted version.

## Funding

This study was supported by grant from the National Natural Science Foundation of Jiangsu Province (BE2018652, BK20201168) and the National Natural Science Foundation of China (81970136).

## Conflict of Interest

The authors declare that the research was conducted in the absence of any commercial or financial relationships that could be construed as a potential conflict of interest.

## Publisher’s Note

All claims expressed in this article are solely those of the authors and do not necessarily represent those of their affiliated organizations, or those of the publisher, the editors and the reviewers. Any product that may be evaluated in this article, or claim that may be made by its manufacturer, is not guaranteed or endorsed by the publisher.

## Supplementary Material

Supplementary Figure 1 Immunophenotypic analysis a primary diagnosis **(A)** and BM morphological analysis after 14 days after ATRA and ATO **(B)**.

Supplementary Figure 2 Sequencing chromatogram of the junction sequences of the THRAP3- RARA fusion transcript (exon 2 of THRAP3 and exon 3 of RARA genes) **(A)** and RARA-THRAP3 fusion transcript (exon 9 of THRAP3 and exon 6 of RARA genes) **(B)**.
