# Supplementary figures and images for: Clinical Response to Venetoclax and Decitabine in Acute Promyelocytic Leukemia With a Novel RARA-THRAP3 Fusion: A Case Report
Source: Front Oncol. 2022 Feb 7;12:828852. doi: 10.3389/fonc.2022.828852 (PMC8858936; doi:10.3389/fonc.2022.828852)

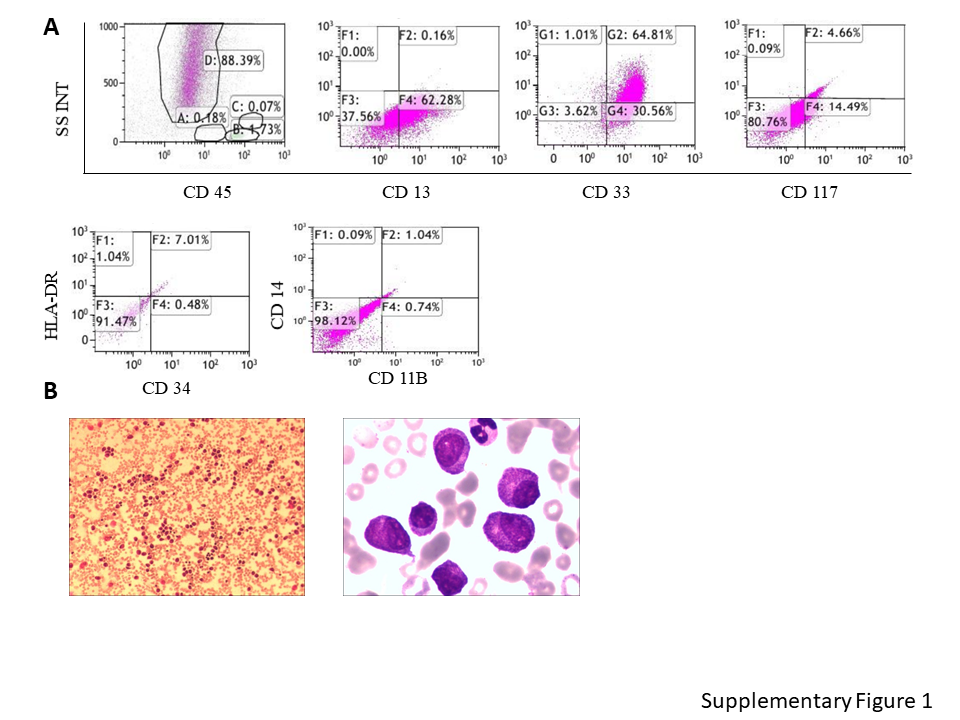

Supplement: Supplementary Figure 1 — Immunophenotypic analysis a primary diagnosis (A) and BM morphological analysis after 14 days after ATRA and ATO (B). [file Image_1.tif]

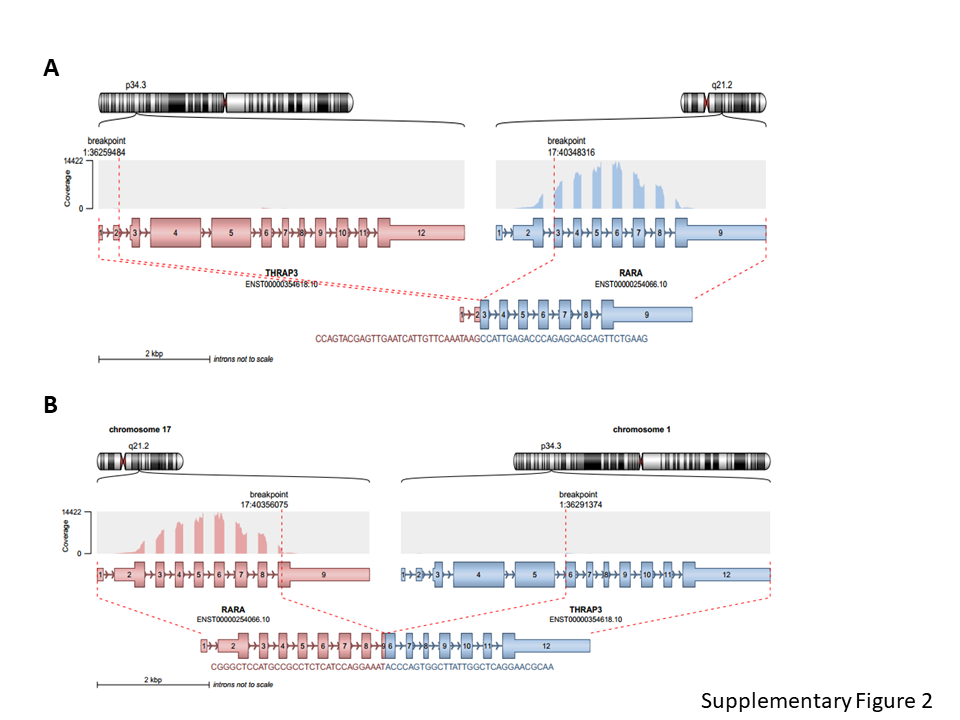

Supplement: Supplementary Figure 2 — Sequencing chromatogram of the junction sequences of the THRAP3- RARA fusion transcript (exon 2 of THRAP3 and exon 3 of RARA genes) (A) and RARA-THRAP3 fusion transcript (exon 9 of THRAP3 and exon 6 of RARA genes) (B). [file Image_2.tif]
